# Opportunities to Digitally Enable Falls Prevention in Older Adults

**DOI:** 10.1093/geront/gnaf016

**Published:** 2025-01-25

**Authors:** Hannah Gulline, Angela Melder, Anna Barker, Marissa Dickins, Karen Smith, Darshini Ayton

**Affiliations:** Health and Social Care Unit, School of Public Health and Preventive Medicine, Monash University, Melbourne, Australia; Health and Social Care Unit, School of Public Health and Preventive Medicine, Monash University, Melbourne, Australia; Silverchain, Melbourne, Australia; Acute and Critical Care, School of Public Health and Preventive Medicine, Monash University, Melbourne, Australia; Silverchain, Melbourne, Australia; Department of Psychiatry at Monash Health, School of Clinical Sciences, Monash University, Melbourne, Australia; Silverchain, Melbourne, Australia; Department of Epidemiology and Preventive Medicine, School of Public Health and Preventive Medicine, Monash University, Melbourne, Australia; Health and Social Care Unit, School of Public Health and Preventive Medicine, Monash University, Melbourne, Australia

**Keywords:** Community-dwelling, Digitally enabled, Falls prevention, Technology

## Abstract

Falls are a serious problem confronting older adults. Evidence demonstrates that multifactorial interventions that target multiple risk factors can reduce falls. However, resource and access constraints affect intervention uptake and sustainability. In comparison, digitally enabled interventions have the potential to provide greater support and convenience whilst being tailored to an individual. Although digital advancements present an opportunity to improve access, scalability, and sustainability, there is limited knowledge on how to digitally enable traditional interventions. In this article, we summarize the academic literature on digital falls prevention and propose future research directions for digital falls prevention. We examine barriers and enablers to digital falls prevention in aged care, although, given the scarcity of evidence, we draw on lessons from other digital healthcare innovations.

Falls are an unsolved problem affecting older people, family caregivers, health and aged care providers, and governments internationally. Falls are the leading cause of emergency department presentations (Shankar et al., 2017) and injury hospitalizations in older adults aged 65 years and older (Australian Institute of Health and Welfare, 2022) and trigger a cascade of poor health outcomes resulting in morbidity and mortality (Vaishya & Vaish, 2020). Research indicates multifactorial interventions—interventions of two or more components tailored to the fall-risk profile of the older person (Hopewell et al., 2018)—more effectively reduce the rate of falls (Montero-Odasso et al., 2022) when compared with single interventions (Hopewell et al., 2018). Although short-term programs may be effective at reducing falls, ongoing intervention is required to maintain benefits (Sherrington et al., 2020). Implementation of multifactorial interventions is resource intensive constraining scalability and sustainability (Close & Lord, 2022). In contrast, the adoption of digital tools provides opportunities to efficiently scale falls prevention interventions (Cao et al., 2022; Chan et al., 2021).

As technology advanced, digital interventions were identified as having potential to improve falls prevention and detection in community-dwelling older adults (Lord & Close, 2018). The Nonadoption, Abandonment, Scale-up, Spread, and Sustainability (NASSS) framework (Greenhalgh et al., 2017, 2020) is an evidence-based, theory-informed, and pragmatic framework, developed to help design, implement, and evaluate digital health or social care interventions. It consists of seven domains that outline aspects to be considered when developing a technology-based intervention. These include details about the condition or illness, the technology, the value proposition, the adopter system (comprising professional staff, patient, and informal caregivers), the organization(s), the wider (institutional and societal) context, and the interaction and mutual adaptation between all these domains over time (Greenhalgh et al., 2017).

The NASSS framework examines the dynamic interplay between the stakeholders, system, and context of a technology-based intervention to provide a nuanced perception of the determinants, processes, and outcomes (Abell et al., 2023). The NASSS framework has been demonstrated to have external validity to map implementation determinants within health settings. A series of complexity assessment tools (CAT) have been created to facilitate utilization of the NASSS framework as it was designed for academic analysis as opposed to as a practical tool (Greenhalgh et al., 2020). The NASSS-CAT tools include a short version for initial interest gauging, a long version for detailed project planning and stakeholder discussion, a short version, a project version for ongoing complexity monitoring, and a semistructured interview version for research or evaluation. Given the utility of the NASSS framework for implementing technological interventions and the opportunity for digital falls prevention, the NASSS framework could be a valuable tool in guiding digital falls prevention strategies.

We set out to examine the evidence about new and emerging digital interventions to prevent falls for community-dwelling older adults. Working in partnership, researchers at Monash University and Silverchain —a large Australian in-home care provider—sought to guide research and practice in this area by answering the following research questions: What digital falls prevention interventions in the community context align with NASSS domains? What can we learn from digital health broadly applied to NASSS to inform digital falls prevention?

## Digital Falls Prevention

A comprehensive evidence-base highlights the importance of exercise in falls prevention amongst older adults, with several options to digitally enable exercise, including exergames, telehealth, and virtual delivery (Ambrens et al., 2022). In Table 1 we provide an overview of identified digital falls prevention interventions for community-dwelling older adults mapped to the first four domains of the NASSS framework and described further below.

**Table 1.** Digital Falls Prevention Interventions Mapped to the Condition, Technology, Value Proposition, and Adopter Domains of the Nonadoption, Abandonment, Scale-Up, Spread, and Sustainability Framework

Digital falls intervention	Condition—Nature of condition or illness, comorbidities, sociocultural influences	Technology—Material features, type of data generated, knowledge needed to use, technology supply model	Value proposition—supply-side value (to developer), demand-side value (to patient/client)	Adopters—staff (role, identity), patient/client (simple vs complex input), carers (available nature of input)
Exergames	-Shown to be beneficial in studies involving healthy older adults but limited research amongst older adults with one or more comorbidities. -Inconclusive evidence on effect of fall history due to limited reporting.	-Requires access to exergame platform, exergame software, and screen. -Requires sufficient technological proficiency to set up and operate. -Many different exergames and platforms are available and most reviews group the different types together—inconclusive evidence of specific exergames.	-Shown to reduce fall risk and improve balance in older adults. -Effectiveness, safety and cost-effectiveness value for older adults have been shown in retirement homes, community centers, hospitals, and other facilities with staff supervision. -Inconclusive effective and cost-effectiveness evidence of value for older adults in home-based settings.	-Older adults must actively engagement with exergame technology. -Shown to be acceptable and user-friendly for older adults. -Older adults need support selecting exergame software/platform and engaging with exergames initially, such as supervision while learning the technology.
Telehealth and Exercise	-Mostly healthy older adults participated in individual studies on falls. -Studies that did not focus on falls involved participants with comorbidities. -Inconclusive evidence on effect of fall history due to limited reporting.	-Requires access to a telecommunication device and stable internet connection. -Requires adequate technological proficiency to operate the telecommunication device.	-Shown to improve older adults exercise technique with health professional’s guidance and adherence to reduce falls risk. -Access to health professionals without needing to travel to a medical facility, which is pertinent for older adults who might fear the spread of infection particular after the COVID-19 pandemic. -Access to services that may not be locally available for all older adults.	-Older adults must actively engage with the telehealth and exercise. -Older adults’ acceptance of telehealth is becoming increasingly prominent, with cost and time savings. -Telehealth interventions have been shown to be acceptable and cost-effective to health professionals.
Virtual exercise program	-Shown to be beneficial in studies involving healthy older adults. -Some reporting on the effect of fall risk but what constituted “fall risk” was undefined.	-Requires access to necessary device and stable internet connection. -May require access to exercise equipment for some virtual exercise programs. -Requires adequate technological proficiency to navigate website or app.	-Shown to be accessible and cost-effective for older adults with the necessary technology. -Access to exercise programs without any travel required from older adults. -Virtual exercise programs can have a social aspect that enhances engagement and reduces social isolation for older adults. -Professionals are able to run virtual exercise programs with reduced expenses compared to in-person exercise programs.	-Older adults must actively engage with virtual exercise programs. -Older adults’ acceptance of technology is increasing, with cost and time savings associated. -The social element of virtual exercise programs may motivate sustained engagement and social connection, which may contribute to adoption by older adults.
Smart home systems	-Shown to be beneficial in studies involving healthy older adults. -Inconclusive evidence on effect of fall history due to limited reporting.	-Requires access to technologies throughout the home. -Following installation by a professional, smart home systems do not require older adults to possess technological proficiency.	-Shown to reduce falls occurrence whilst facilitating older adults to remain independent within their home. -Procurement, installation, and maintenance costs can be significant and might outweigh the value of the reduced falls to older adults.	-Smart home systems can be designed to reduce fall risk through automated processes that do not require active engagement from older adults or behavior/lifestyle changes. -Older adults must have a stable home environment that can be modified as necessary to install technologies.
Falls detection systems (ambient, wearable, hybrid)	-Reviews focused on the technology rather than falls. -The technology was tested in adults of all ages.	-Requires access to sensors. -Requires access to software and someone with a device that can receive an alert if the sensors detect a fall.	- Shown to detect falls while facilitating older adults to remain independent within their home and/or community—different systems have different value depending on the older adult’s lifestyle. -Some older adults may feel this value is outweighed by the belief these sensors compromise their privacy. -Provide security to family/caregivers knowing they will be notified of falls and help if needed.	-Ambient fall detection systems and sensors require passive input from older adults. -Fall detection systems with wearable sensors require active engagement in terms of older adults keeping the sensors on their person and charging the battery as required. -Family/caregivers must be prepared for falls and act when an alert is triggered. -Fall detection systems can be expensive and may prohibit adoption by older adults.

### Exergames

Exergames combine exercise interventions with the interactive and adaptive elements of video games (Nawaz et al., 2015). Two systematic reviews and meta-analyses demonstrated that exergames lead to a 9% reduction in falls incidence for at-risk older adults and improved dynamic balance (Chen et al., 2021; Lapierre et al., 2023). Additionally, exergames have been demonstrated to be safe (Alhagbani & Williams, 2021), acceptable with mostly positive attitudes, engagement, and satisfaction, and user-friendly when the game is designed for older adults (Buyle et al., 2022; Ning et al., 2022). Research on exergames typically employs the Wii exergame platform and occurs in facilities (e.g., hospitals, rehabilitation facilities, retirement homes, and community centers) with supervision by staff, which enhances the cost–benefit (Afridi et al., 2021; Alhagbani & Williams, 2021; Ge et al., 2022; Stanmore et al., 2019). Further research is required to examine exergame effectiveness in an unsupervised, home-based setting and the cost implications of this approach (Afridi et al., 2021; Alhagbani & Williams, 2021).

### Telehealth and Exercise

Telehealth and exercise utilize telecommunication to provide support and advice to foster continued participation in exercise (Chan et al., 2021). This enables health professionals to virtually assess older adults and offer advice and encouragement, enhancing adherence and improving performance (Chan et al., 2021; Zhang et al., 2023). Evidence indicates that this combination significantly improved balance by 62% (Chan et al., 2021). Whilst was not being statistically significant, the reduced falls risk of 16% was similar to that of face-to-face exercise programs (Chan et al., 2021).

### Virtual Exercise Program

Virtual exercise programs utilize websites and apps to support, motivate, and monitor older adults’ participation in exercise. Individuals are guided via videos, oral directions, and written instructions (van het Reve et al., 2014). The “virtual gym” requires an electronic device and exercises are tailored to individual needs and ability, with difficulty increases aligned to participant’s improvements (Ambrens et al., 2022; Chan et al., 2021). Some virtual exercise programs require equipment (e.g., stepping box) that is supplied (Ambrens et al., 2022). Motivational messages may also be included, whereby participants interact with trainers and/or other individuals engaging in exercise via online communities (Chan et al., 2021; Silveira et al., 2013). Virtual exercise programs facilitate balance and strength exercises in an accessible, convenient, and cost-effective way (McGarrigle et al., 2020). The social aspect of virtual exercise programs has been shown to enhance engagement and enjoyment (Baez et al., 2017; Chan et al., 2021) and addresses social isolation in older adults (Chen & Schulz, 2016). In summary, the benefits of these digitally enabled falls prevention exercises are threefold: personalized exercises, motivational support, and socialization (Chan et al., 2021).

### Smart Home Systems

Smart home systems encompass a variety of innovative technologies including ambient assisted living, ambient intelligence, artificial intelligence, partial assistive technologies, information and communication technology, and the Internet of Things (Marikyan et al., 2019). For older adults, the purpose of these technologies is to improve personal safety, monitor health, expand social interactions, and increase living environment control (Johnny & Leung, 2016). A smart home system can facilitate the detection and elimination of environmental hazards (Chan et al., 2021). For example, an automated guiding light to prevent falls during the night (Thölking et al., 2020).

### Falls Detection Systems

Falls detection and prevention systems consist of sensors and a monitoring device to detect changes in movement that may indicate a fall and trigger an alert to a caregiver/family member that assistance may be required (Torres-Guzman et al., 2023). This identification of changes can prompt proactive falls risk review and implementation of prevention strategies. These systems provide increased safety and reassurance for older adults to continue living independently in the community (Torres-Guzman et al., 2023). Falls detection and prevention systems can be classified as either ambient, wearable, or a hybrid system (Singh et al., 2020).

#### Ambient systems

An ambient system employs cameras and stationary sensors to monitor an individual (Usmani et al., 2021). However, this type of system has limited coverage, with no protection for the older adult when external to the observation area (Usmani et al., 2021). Ambient systems also have potential blind spots and are traditionally expensive to install (Singh et al., 2020). Older adults may dislike feeling observed and their privacy compromised (De Miguel et al., 2017; Torres-Guzman et al., 2023). Therefore, ambient falls detection systems may not be feasible and/or acceptable for all older adults.

#### Wearables

Wearables utilize motion sensors attached to individuals, such as gyroscopes and accelerometers, to monitor falls and recognize changes in risk factors (e.g., sleep patterns and blood pressure) that may predict future falls (Rucco et al., 2018; Sheikh et al., 2021). Unlike ambient systems, wearable device usage is not confined to a specific location, making them more feasible for activities of daily life. They are more affordable than ambient systems. However, wearables are limited by device battery life (Rault et al., 2017), lifetime processing power, and the requisite algorithm to interpret data to identify falls, with many generating false alarms frequently (Usmani et al., 2021). Moreover, wearable device usefulness is restricted by whether the user wears them regularly (Singh et al., 2020).

An alternative to traditional wearable devices is to capitalize on sensors integrated into smartphones (Torres-Guzman et al., 2023). Many older adults own a smartphone, making it an accessible, cost-effective, and acceptable option with users more likely to have it with them while carrying out activities of daily life compared to other sensors (Torres-Guzman et al., 2023). Further research is required to test falls detection ability according to smartphone position (Torres-Guzman et al., 2023) and to validate the use of smartphones against gold-standard methods, particularly in older adult populations with greater falls risk (Roeing et al., 2017). Technological advances are necessary to enhance usability, such as improving power consumption to enable the battery to last throughout the day (Stampfler et al., 2022). Like other wearables, smartphone sensors are limited by whether the device is on the person. There are times whilst at home when a device may not be on the person, such as during the night, rendering them incapable of detecting falls at this time.

#### Hybrid fall detection systems

Hybrid fall detection systems utilize different ambient and/or wearable sensors to significantly improve falls detection capabilities (Singh et al., 2020). Whilst these systems have the potential for improved reliability and minimizing gaps in coverage, compromise is needed between sensitivity and accuracy. Increased sensitivity detects low-impact falls but prompts false alarms, whereas highly accurate systems may overlook some fall events (Singh et al., 2020). This novel approach requires further research. The additional sensors and the computational costs to fuse and process the extensive data come at greater expense than a simpler system (Koshmak et al., 2016), making them an unrealistic option for many community-dwelling older adults.

## Digitally Enabling Multifactorial Interventions—What Are the Benefits?

Systematic reviews and meta-analyses have demonstrated that digital approaches to falls prevention enhanced adherence, objective monitoring, and older adults motivation through feedback, education, guidance, and remote support (Hughes et al., 2018). Many studies employed composite strategies to enhance adherence suggesting that a multifactorial approach to adherence, as well as multifactorial interventions, may be appropriate to optimize effectiveness (Hughes et al., 2018). Technology has the potential to offer more support than traditional approaches, with a recent study comparing a digital and paper-delivered exercise program finding that the digital group reported a significantly higher degree of feeling supported and content (Mansson et al., 2020). Digitally enabled care can be more convenient than traditional interventions as it can be delivered in homes without distance restricting access (Yang et al., 2022) and has great capacity to be personalized to an individual’s abilities, needs, and preferences, which may increase adoption amongst older adults (Fabbrizio et al., 2023).

Older adults are increasingly technologically literate with interventions to maintain independence, improve their quality of life and support a healthy lifestyle (Kaur & Chen, 2022). However, a technology’s effectiveness for this population is contingent on the usability of the interface, with many physical and cognitive training applications unsuitable for older adults (Kaur & Chen, 2022). Usability must be considered during software development to ensure it is appropriate for older adults with diverse cognitive and physical abilities. Many applications and websites for falls prevention exercises exist, but knowledge of the effectiveness of these specific programs and the amount of support and motivational elements required to support online delivery is limited (McGarrigle et al., 2020).

Above we have outlined emerging digital strategies, however, recent reviews have not captured the effectiveness of digital or technology-based elements within multifactorial falls prevention. Furthermore, there is limited understanding of implementation strategies of digitally enabled interventions for sustainment, scalability, and adherence. This paucity of research and knowledge means we need to consider comparable interventions to provide insights into implementation and impact in real-world settings, including recent learning from the coronavirus disease 2019 (COVID-19) pandemic.

## Lessons Learnt From Other Digital Interventions for Older Adults

The spread, scale-up, and sustainability of video consulting in healthcare in response to COVID-19 revealed mixed results of early adoption, acceptability, safety, and effectiveness for the management of health conditions (James et al., 2021). The enablers of spread and scale-up included operational elements, such as reimbursement mechanisms, user-friendly technology, ability to adapt technology and service models, and leadership elements such as embedded leadership, leveraging preexisting staff relationships, and the role of champions (James et al., 2021). Challenges were related to service development, including absence of long-term strategic planning, resistance to change, cost and reimbursement, the technical experience of staff and limited articulation of scale-up and spread of video consultations complications (James et al., 2021). Overall, the review reported a lack of evidence that can support the spread and scale-up of video consulting (James et al., 2021); however, with emerging evidence from COVID, this is likely to change (Getachew et al., 2023).

Another technological advance in healthcare delivery is the virtual healthcare/hospital model, enabling healthcare organizations to deliver care at a distance (Li et al., 2021; Merchant & Aprahamian, 2022). It is achieved by delivering care through a variety of “tele-modalities” such as automated device-based telemonitoring, mobile telemonitoring, interactive voice response technologies, video-consultation, and web-based monitoring (Moore et al., 2020). It includes virtual wards (supported by tele-modalities) and hospitals in-the-home supported by healthcare professionals delivering services within the community (Man Qing et al., 2021; Moore et al., 2020). Evaluations of these interventions have happened at a clinical and system level for a range of health conditions. Benefits identified include improvements in clinical outcomes and health system outcomes such as reduced hospitalization rates (Li et al., 2021; Savira et al., 2023). Implementation challenges with virtual healthcare include determining who is delivering care and leveraging workforce strengths, coordination and integration of care delivery, optimizing person-centered care delivery and data and clinical governance (Jessup et al., 2022; Norman et al., 2023). Lessons gleaned from these and other comparable interventions should be tailored and incorporated into future digital falls prevention strategies.

## Next Steps for Falls Prevention Research

The remaining domains of the NASSS framework—organization, wider system, and embedding adaption over time are vital to sustain the digital revolution of falls prevention in community aged care. In our examination of the evidence, these domains were rarely included. Key areas for further exploration and development include:

- Target population selection—do participants have a high or low risk of falling? Often interventions targeted healthy older adults with low falls risk. Falls prevention interventions need to target individuals with higher risk of falling to reduce the rate of falls.
- Technological proficiency—how proficient are older people and what resources are required to support adaptation to changing devices and technology (Schroeder et al., 2023)?
- Cost-effectiveness and equity of access to technology—how will the integration of technology either widen or close gaps in equity. Unequal access across population groups has implications for digital health inequities (Kepper et al., 2024). Additionally, the cost of updating technology has sustainability implications both for providers and patients/clients.

## Conclusion

We set out to examine the evidence about new and emerging digital interventions to prevent falls and considered their feasibility for community-dwelling older adults. Whilst evidence of individual digital intervention’s utility in preventing falls exists, digital enablement’s effectiveness in multifactorial interventions is inconclusive. Further research is required to provide clarity on the effectiveness and feasibility of digitally enabled multifactorial interventions.

Additionally, we see an absent or poorly developed body of evidence informing digital falls prevention implementation. However, we can gain insights from other successful technology-based interventions such as the virtual hospital and telehealth innovations. Future research needs to examine the integration of interventions into activities of daily living to improve adherence. With an extended focus on how organization, community, and policy-level interventions (and implementation of them) can foster sustainment, equity, and scale-up opportunities. Applying valuable insights from implementation science reveals a clear pathway to expanding and guiding falls prevention research.

## Data Availability

Authors do not report data and therefore the preregistration and data availability requirements are not applicable.
